# Conventional Amphotericin B Associated Nephrotoxicity in Patients With Hematologic Malignancies

**DOI:** 10.7759/cureus.16445

**Published:** 2021-07-17

**Authors:** Vildan Gursoy, Fahir Ozkalemkas, Vildan Ozkocaman, Zafer Serenli Yegen, Ibrahim Ethem Pinar, Beyza Ener, Halis Akalın, Esra Kazak, Ridvan Ali, Alparslan Ersoy

**Affiliations:** 1 Division of Hematology, Department of Internal Medicine, Medical School of Usak University, Usak, TUR; 2 Division of Hematology, Department of Internal Medicine, Uludag University Medical Faculty, Bursa, TUR; 3 Division of Hematology, Bursa City Hospital, Bursa, TUR; 4 Department of Microbiology, Uludag University Medical Faculty, Bursa, TUR; 5 Department of Infectious Diseases and Clinical Microbiology, Uludag University Medical Faculty, Bursa, TUR; 6 Division of Nephrology, Department of Internal Medicine, Uludag University Medical Faculty, Bursa, TUR

**Keywords:** conventional amphotericin b, nephrotoxicity, hematological malignancy, hypokalemia, fungal infections

## Abstract

Introduction: Amphotericin B (AmB-d) is one of the most effective therapeutic options against frequently life-threatening systemic fungal infections in patients with hematologic malignancies. However, significant adverse effects including nephrotoxicity associated with its use limit its more widespread use. The objectives of our study were to determine the incidence of AmB-d associated nephrotoxicity, to evaluate clinical and epidemiological characteristics of patients, and to support the notion that conventional amphotericin B remains a valid therapeutic option among hematologic patients with proper patient selection.

Materials and methods: A total of 110 patients with hematologic malignancies were admitted to our Hematology Unit between January 2014 and November 2017 who required anti-fungal therapy during intensive systemic chemotherapy. The incidence of AmB-d associated nephrotoxicity, side effect profile, time to nephrotoxicity, and clinical and epidemiological characteristics associated with treatment success were assessed retrospectively.

Results: Of the 110 patients receiving AmB-d, 70 (63.6%) were male and 40 (36.4%) were female. The mean age of participants was 44 years. The most common diagnosis was acute myeloid leukemia (n=53, 48.2%), and the most common chemotherapy protocol was 7 + 3 remission-induction (cytarabine 100 mg/m² days 1-7, Idarubicin 12 mg/m² days 1-3; n=24, 21.8%). In 56.4% of the patients, antifungal therapy was given empirically. In 40 patients (36.4%), nephrotoxicity was observed following antifungal treatment, and only four patients had stage 3 renal failure. The mean duration of time to nephrotoxicity from initiation of amphotericin B was four days (min: 2, max: 31). All patients were found to receive at least one additional potential nephrotoxic treatment during the antifungal treatment process.

Conclusion: AmB-d is associated with a significant risk of nephrotoxicity. In most hematological patients, antifungal treatment is initiated empirically, and patients received prolonged courses of treatment. Therefore, it is plausible to initiate such treatment with AmB-d, when one considers the already high treatment costs in this patient group as well as the fact that AmB-d offers similar efficacy to antifungal agents at a lower cost. AmB-d may be recommended as a first-line agent in this patient group with the introduction of newer and more costly antifungal agents when needed, on the basis of the fact that these patients can be closely monitored in a hospital setting, reversible nature of nephrotoxicity upon discontinuation, and rare occurrence of severe renal failure requiring dialysis.

## Introduction

Despite recent therapeutic advances, severe fungal infections remain an important cause of morbidity and mortality in hematology units. The traditional amphotericin B was described in the mid-1950s as the first anti-fungal agent effective against systemic mycoses [1]. Amphotericin-B (AmB-d) deoxycholate, the oldest polyene class of drugs, has been approved for use in the treatment of invasive fungal infections and has been considered as a “gold standard” drug since the 1960s [2,3]. The efficacy and safety of newly developed antifungal agents are tested against this agent [4]. AmB-d exhibits a wide spectrum of activity against a number of microorganisms including *Aspergillus fumigatus*, *Blastomyces* *dermatitidis*, Candida spp., *Coccidioides immitis*, *Cryptococcus neoformans*, *Histoplasma capsulatum*, and *Paracoccidioides​​​​​​​ brasiliensis* [5]. However, toxicity associated with the use of AmB-d, and particularly nephrotoxicity, is a major factor limiting its clinical utility [6]. Important characteristics of AmB-d associated nephrotoxicity include increased serum creatinine, reduced glomerular filtration rate (GFR), urinary loss of potassium, hypokalemia, urinary loss of magnesium, and hypomagnesemia [7].

Due to these toxicities, the role of other antifungal agents as an alternative to AmB-d has been evaluated for empirical treatment. Lipid formulations have been shown to provide similar efficacy to AmB-d, with a reduced risk of nephrotoxicity [8,9]. However, these formulations are more costly as compared to the conventional forms. Since these preparations generally require higher doses than AmB-d, the cost is even higher. In developing countries such as Turkey, treatment costs are an important consideration when choosing therapeutic agents due to the prolonged hospital stay among patients with hematologic malignancies. A cost-effective treatment with minimal risks may be possible by proper patient selection and management.

In this study, our objectives were to determine AmB-d associated nephrotoxicity incidence among patients with hematologic malignancies who frequently require antifungal treatment, to evaluate risk factors in patients who developed nephrotoxicity, and to discuss the utility of this agent providing similar efficacy at a lower cost compared to other agents in properly selected patients.

## Materials and methods

A total of 110 patients with hematologic malignancies who were admitted to the Adult Hematology Unit between January 2014 and November 2017 and who received intensive systemic chemotherapy and required anti-fungal treatment during that period were included in this study. Patients with AmB-d associated nephrotoxicity, epidemiologic data, clinical characteristics, and hematologic parameters were retrieved from the electronic database. Also, the following parameters were retrospectively assessed: demographic data such as age and gender, primary hematological diagnosis, chemotherapy regimen(s) administered, duration of expected neutropenia secondary to treatment, patient-related risk factors, concomitant nephrotoxic agents, laboratory parameters, creatinine (baseline, maximum, and most recent), stage of the renal failure, history of recurrent admission, indications, and duration of use for AmB-d, hepatotoxicity secondary to AmB-d use, metabolic abnormalities, discharge status, death, and transplantation.

Renal failure was staged according to AKIN (Acute Kidney Injury Network) criteria, which classifies acute kidney injury (AKI) in three stages of severity. (stages 1, 2, and 3) based on levels of serum creatinine and the patient’s urine output. Accordingly, stage 1 injury was diagnosed with a ≥0.3 mg/dl or 1.5-2 fold increase in baseline creatinine level (or urinary output for 6-12 hours <0.5 ml/kg/hour); stage 2 injury was diagnosed with >2-3 fold increase in baseline serum creatinine (or ≥12-hour urinary output <0.5 ml/kg/hour); and stage 3 increase was diagnosed with >3-fold increase in baseline serum creatinine (or 24-hour urinary output <0.3 ml/kg/hour or anuria ≥12 hours). Hypokalemia was considered mild when serum potassium level was 3 to 3.4 meq/L, and severe when serum potassium level was <3 meq/L. The cut-off values for mild and severe hypomagnesemia were 1 to 1.7 mg/dl, and <1 mg/dl, respectively.

Exclusion criteria were age <18 years, absence of AmB-d treatment, treatment on an outpatient basis, presence of kidney impairment at admission, known history of chronic renal disease, and missing data. The study was conducted in accordance with the Declaration of Helsinki and was approved by the Ethics Committee of the Medical Faculty of Bursa Uludağ University (February 19, 2020; no: 2020-3/1).

Statistical analyses

The normal distribution of the variables was assessed with the Shapiro-Wilk test. Presentable with normal distribution were expressed as mean ± standard deviation, and comparison of two independent groups were performed using the independent sample t-test. Presentable without normal distribution were expressed as median values (minimum-maximum), and the two independent groups were compared with the Mann-Whitney U test. Categorical data were described with frequency and percentage (n,%) and were compared with Pearson's chi-square test, Fisher’s exact test, and Fisher-Freeman-Halton test. Statistical analyses were performed using the IBM SPSS Statistics 22.0 software pack (IBM Corp., Armonk, NY), at a significance level of p=0.05.

## Results

During the three-year study period, a total of 110 patients with hematological malignancies who were admitted to the Hematology Unit, Medical Faculty of Bursa Uludag University and treated with AmB-d as an anti-fungal regimen were recruited. Of these 110 patients, 62.7% (n=69) were male and 37.3% (n=41) were female, with an overall median age of 44 ± 14.97 years. Two groups of patients were defined on the basis of age ≤45 years (n=56, 51.4%), and >45 years (n=54, 48.6%). The most common presentations included acute myeloid leukemia (n=53, 48.2%), acute lymphoid leukemia (n=35, 31.2%), and non-Hodgkin lymphoma (n=12, 11%). Most cases of antifungal treatments were required in the course of intensive induction treatments such as 7 + 3 remission-induction (n=24, 21.8%; cytarabine, Idarubicin), Hyper-cvad (n=23, 20.9%; cyclophosphamide, vincristine, doxorubicin, cytarabine, methotrexate, and dexamethasone), and Flag-ida (n=20, 18.2%; fludarabine, cytarabine, idarubicine). In 87.3% of the patients (n=96), the expected duration of neutropenia secondary to treatment was more than 10 days. In 60% of the patients (n=66), steroids were administered, both as a part of the chemotherapeutic regimen and also as a premedication prior to AmB-d treatment. History of allogeneic transplantation was present in 10.9% of the patients (n=12), while 64.5% (n=71) had a history of at least two hospital admissions. Of the recurrent admissions, 50% (n=55) had occurred within the past six months. Among patients requiring antifungal treatment, 31.8% (n=35) had at least one comorbid disease such as diabetes, hypertension, obesity, coronary artery disease, or hepatitis carrier condition (Table 1).

**Table 1:** Patient characteristics AA: aplastic anemia, AML: acute myeloid leukemia, ALL: acute lymphoblastic leukemia, NHL: non-Hodgkin lymphoma, MDS: myelodysplastic syndrome, CLL: chronic lymphocytic leukemia, HCL: hairy cell leukemia, MM: multiple myeloma, CML: chronic myeloid leukemia, HL: Hodgkin lymphoma.

Gender		Median age% (age range) 44% (19–76)	
Female	37.3% (n=41)	Female	42.9% (20–69)
Male	62.7% (n=69)	Male	44.7% (19–76)
Hematologic malignancy		CTx protocol administered	
AML	48.2% (n=53)	7 + 3 remission induction (cytarabine 100 mg/m^2 ^days 1–7; idarubicine 12 mg/m^2 ^days 1–3)	21.8% (n=24)
ALL	31.8% (n=35)	Hyper-cvad (cyclophosphamide, 300 mg/m^2^ days 1–3; vincristine, 2 mg days 4 and 11; doxorubicin 50 mg/m^2^ day 4; and dexamethasone 40 mg days 1–4 and 11–14. Courses 2, 4, 6, and 8 consisted of methotrexate, 1 g/m^2 ^day 1; and cytarabine, 3 g/m^2^ days 2 and 3)	20.9% (n=23)
NHL	11 % (n=12)	FLAG-ida (fludarabine 30 mg/m^2^ days 1–5; cytarabine 2 g/m^2^ days 1–5; idarubicin 10 mg/m^2 ^days 1–3)	18.2% (n=20)
MDS	1.8% (n=2)	EMA (mitoxantrone 12 mg/m² days 1–3; etoposide 200 mg/m² days 8–10 and cytarabine 500 mg/m² days 1–3 and 8–10)	12.8% (n=14)
CLL	1.8% (n=2)	Consolidation (cytarabine 6 g/m^2^ days 1, 3, 5)	11.8% (n=13)
HCL	1.8% (n=2)	Azacitidine (50mg/m^2^ days 1–10 or 75mg/m^2^ days 1–7)	1.8% (n=2)
MM	0.9% (n=1)	DHAP (dexamethasone 40 mg days 1–4, cytarabine 2000 mg/m^2^/12 hours day 2, cisplatin 100 mg/m^2^ day 1)	1.8% (n=2)
CML	0.9% (n=1)	R-FC (rituximab 375-500 mg/m^2 ^day 1; fludarabine 25 mg/m^2^ days 1–3 and cyclophosphamide 250 mg/m^2^ days 1–3)	0.9% (n=1)
AA	0.9% (n=1)	Cladribine (0.14 mg/kg days 1–5)	0.9% (n=1)
HL	0.9% (n=1)	Other regimens	5.5% (n=6)
		Patients without CTx	3.6% (n=4)
Transplantation		Steroid use	
Yes	10.9% (n=12)	Yes	60% (n=66)
No	89.1% (n=98)	No	40% (n=44)
History of admission		History of recurrent admission	
First	35.5% (n=39)	Within <6 months	50% (n=55)
Second	15.4% (n=17)	>6 months	14.5% (n=16)
Third or more	49.1% (n=54)		
Expected duration of neutropenia in association with the chemotherapy regimen		Risk factor	
>10 days	87.3% (n=96)	At least one	31.8% (n=35)
<10 days	12.7% (n=14)	None	68.2% (n=75)

Indications for AmB-d use included empiric treatment (fever, neutropenia, or other causes) in 56.4% (n=62), documented fungal infection (microbial growth in blood, urine, tissue, central nervous system, sputum, or other cultures) in 19.1% (n=21), and presence of radiological findings (thorax CT image suggestive of invasive pulmonary Aspergillosis) in 24.5% (n=27).

The mean weight of patients was 74.1 ± 13.32 kg (min: 40, max:112 kg), and the mean body surface area was 1.81 ± 0.18 m^2^ (min: 1.4, max: 2.2 m2). AmB-d infusions were administered over a two to six-hour period. The median daily dose was 70 mg (40-100; mean 71.9 ± 13.03 mg), with a cumulative dose of 480 mg (50-2250; mean 712.0 ± 582.3). The median duration of AmB-d treatment was seven days (1-45 days; mean 10.5 ± 9.35 days). A cumulative dose exceeding 1 g was given in 28.2% (n=31) of the patients. Duration, daily dose, and cumulative dose of AmB-d treatment were not significantly different between sexes (p>0.05). On the other hand, the duration and cumulative dose of AmB-d treatment were significantly higher among those ≤45 years of age (p<0.05). The age groups were comparable with respect to daily AmB-d dose.

The laboratory parameters at the time of admission were as follows: white blood cell (WBC) 7.6 × 10^3^/µL (min 0.1 - max 662), neutrophil (neu) 2.6 × 10^3^/µL (min 0.01 - max 68.9), hemogobin (hgb) 10.2± 2.2 g/dl, platelets (plt) 62.2 × 10^3^/µL (min 4.2 - max 570), urea: 30 mg/dl (min 9 - max 95), creatinine 0.7 mg/dl (min 0.4 - max 3.8), sodium 138 mmol/L (min 122 - max 144), potassium 3.9 meq/L (min 2 - max 5.7), calcium 8.5 mg/dl (min 7.2- max 10.4), aspartate aminotransferase (AST) 28 U/L (min 7-max 194), alanine aminotransferase (ALT) 26.5 U/L (min 6-max 315), and lactic dehydrogenase (LDH) 316.5 U/L (min 94- max 4500). Pre-treatment baseline creatinine was 0.69 ± 0.13 mg/dl (median 0.7, min: 0.4-max:1.08), and post-treatment final creatinine was 0.9 ± 0.4 mg/dl (median 0.8; min: 0.4-max: 2.24), with highest creatinine level of 1.01 ± 0.42 mg/dl (median 0.9; min: 0.5-max:2.25) recorded during treatment. While there were significant gender differences in terms of baseline and highest creatinine levels (p < 0.01), the final post-treatment creatinine was similar across the sexes. Also, there were significant differences in terms of creatinine between age groups (p<0.05), while the final and maximum creatinine levels were comparable between the groups. Baseline and highest creatinine did not differ between patients with at least one risk factor (diabetes, hypertension, coronary artery disease, etc.) and patients with no risk factor (Table 2).

**Table 2:** Amphotericin B nephrotoxicity according to gender, age, and risk factors

	Basal creatinine	Final creatinine	Highest creatinine
Overall	0.69±0.13	0.80 (0.4–2.24)	0.90 (0.50–2.25)
Gender			
Female (n=41)	0.64±0.12	0.70 (0.40–2.04)	0.70 (0.50–2.04)
Male (n=69)	0.72±0.13	0.86 (0.46–2.24)	1.00 (0.54–2.25)
P-value	0.001	0.008	0.001
Age			
≤45 (n=56)	0.66±0.13	0.70 (0.40–2.24)	0.90 (0.53–2.25)
>45 (n=54)	0.72±0.13	0.82 (0.46–2.04)	0.90 (0.50–2.04)
P-value	0.018	0.107	0.917
Risk factors			
Absent (n=75)	0.69±0.12	0.80 (0.43–2.24)	0.86 (0.50–2.25)
Present (n=35)	0.69±0.15	0.81 (0.40–2.04)	0.92 (0.53–2.12)
P-value	0.968	0.345	0.404

Of the 110 patients included, no AmB-d nephrotoxictiy was found in 63.6% (n=70), while renal failure was observed during treatment in 36.4% (n=40). Of these latter group of patients, 75% (n=30) were male. Stages 1, 2, and 3 renal failure were found in 20.9% (n=23), 11.8% (n=13), and 3.7% (n=4) of the patients, respectively (Table 3). Among patients with renal failure, the cumulative dose of AmB-d was <0.5 g in 57.5%, 0.5 to 1 g in 17.5% (n=7), 1 to 1.5 g in 12.5% (n=5), 1.5 to 2 g in 7.5% (n=3), and > 2 g in 5% (n=2). The average time to development of nephrotoxicity after initiation of AmB-d treatment was four days (min: 2, max: 31).

**Table 3:** Side effects associated with AmB-d treatment

Acute renal failure	Total	Female	Male	
Absent	63.6% (n=70)	75.6% (n=31)	56.5% (n=39)	P=0.098
Present	36.4% (n=40)	24.4% (n=10)	43.5% (n=30)	
Stage I	20.9% (n=23)	9.8% (n=4)	27.5% (n=19)	
Stage II	11.8% (n=13)	9.8% (n=4)	13.1% (n=9)	
Stage III	3.7% (n=4)	4.8% (n=2)	2.9% (n=2)	
Hypomagnesemia				
Absent	41.8% (n=46)	36.6% (n=15)	44.9% (n=31)	P=0.15
Present	58.2% (n=64)	63.4% (n=26)	55.1% (n=38)	
<1 mg Severe	1.8% (n=2)	4.9% (n=2)	0% (n=0)	
1–1.7 mg Mild	56.4% (n=62)	58.5% (n=24)	55.1% (n=38)	
Hypokalemia				
Absent	30% (n=33)	24.4% (n=10)	33.3% (n=23)	P=0.306
Present	70% (n=77)	75.6% (n=31)	66.7% (n=46)	
<3 severe	37.3% (n=41)	46.3% (n=19)	31.9% (n=22)	
3–3.4 mild	32.7% (n=36)	29.3% (n=12)	34.8% (n=24)	
Hepatotoxicity				
Absent	89% (n=87)	88.1% (n=32)	79.7% (n=55)	P=1.00
Present	21% (n=23)	21.9% (n=9)	20.3% (n=14	

All patients received at least one potential nephrotoxic drug during the anti-fungal treatment, including acyclovir, methotrexate, vancomycin, loop diuretics (furosemide), and aminoglycosides (amikacin).

The reasons for discontinuation of AmB-d therapy included completion of treatment, patient discharge or death in 42.7% (n=47), allergic reactions in 21% (n=23), development of renal failure in 19.1% (n=21), switching to another effective treatment in 9.1% (n=10), hepatotoxicity in 5.5% (n=6), and severe hypokalemia resistant to replacement treatment in 2.7% (n=3).

Table 4 shows the distribution of renal failure patients according to risk factors, chemotherapy protocol, history of recurrent admissions, history of transplantation, and steroid use. Based on these models, none of these variables were significantly associated with the risk of AmB-d-related nephrotoxicity.

**Table 4:** Comparisons regarding renal failure

	Absent	Stage I	Stage II	Stage III	Renal failure	P-value
Risk factor						0.430
At least one risk factor	31.8% (n=35)	27.1% (n=19)	39.1% (n=9)	46.2% (n=6)	25% (n=1)	
None	68.2% (n=75)	72.9% (n=51)	60.9% (n=14)	53.8% (n=7)	75% (n=3)	
Chemotherapy protocol given						
7+3 remission induction	21.8% (n=24)	21.4% (n=15)	26.1% (n=6)	7.7% (n=1)	50% (n=2)	
Hyper-cvad	20.9% (n=23)	18.6% (n=13)	21.7% (n=5)	38.5% (n=5	0% (n=0)	
Flag-ida	18.2% (n=20)	20% (n=14)	17.4% (n=4)	15.4% (n=2)	0% (n=0)	
EMA	12.8% (n=14)	17.1% (n=12)	0% (n=0)	7.7% (n=1)	25% (n=1)	
Consolidation	11.8% (n=13)	8.6% (n=6)	17.4% (n=4)	23.1% (n=3)	0% (n=0)	
Vidaza	1.8% (n=2)	1.4% (n=1)	4.4% (n=1)	0% (n=0)	0% (n=0)	
DHAP	1.8% (n=2)	1.4% (n=1)	4.4% (n=1)	0% (n=0)	0% (n=0)	
R-FC	0.9% (n=1)	1.4% (n=1)	0% (n=0)	0% (n=0)	0% (n=0)	
Cladribine	0.9% (n=1)	1.4% (n=1)	0% (n=0)	0% (n=0)	0% (n=0)	
Other regimens	5.5% (n=6)	4.3% (n=3)	4.4% (n=1)	7.7% (n=1)	25% (n=1)	
Patients without CTx	3.6% (n=4)	4.3% (n=3)	4.4% (n=1)	0% (n=0)	0% (n=0)	
Admission history						0.615
First	35.5% (n=39)	32.9% (n=23)	34.8% (n=8)	38.5% (n=5)	75% (n=3)	
Second	15.5% (n=17)	17.1% (n=12)	21.7% (n=5)	0% (n=0	0% (n=0)	
Third or more	49% (n=54)	50% (n=35)	43.5% (n=10)	61.5% (n=8)	25% (n=1)	
Transplantation	10.9% (n=12)	11.4% (n=8)	4.4% (n=1)	15.4% (n=2)	25% (n=1)	
No transplantation	89.1% (n=98)	88.6% (n=62)	95.6% (n=22)	84.6% (n=11)	75% (n=3)	
Steroid use						0.302
Yes	60% (n=66)	62.9% (n=44)	43.5% (n=10)	69.2% (n=9)	75% (n=3)	
No	40% (n=44)	37.1% (n=26)	56.5% (n=13)	30.8% (n=4)	25% (n=1)	

In 52.5% (n=21) of the 40 patients who developed nephrotoxicity, the treatment was discontinued due to nephrotoxicity, while the reasons for treatment discontinuation in the remaining 19 patients were hepatotoxicity in one, switch to other effective treatments in two, discharge in seven, switch to oral therapy in two, adequate treatment of the causative agent in three, and death in four. In this patient group, the mean duration of medication use was seven days (2-45).

Toxicities other than AmB-d-related renal failure were also assessed. Among these, the most common toxic effects included hypokalemia, hypomagnesemia, and hepatotoxicity. In 70% (n=77) of the 110 patients, hypokalemia was detected, which was severe (potassium <3 mEq) in 37.3% (n=41) requiring potassium replacement. Hypomagnesemia was found in 58.2% (n=64) of the patients, with only two patients experiencing severe hypomagnesemia (<1 mEq). On the other hand, 21% (n=23) of the patients had hepatotoxicity (Table 3), being grade I (AST/ALT≤2.5 x N, bil:N) in seven, grade II (AST/ALT: 2.6-5 x N, bil:<1.5 x N) in six, grade III (AST/ALT: 5.1-20 x N, bil:1.5 -3 x N) in seven, and grade IV (AST/ALT: >20 x N, bil:>3 x N) in three.

Of the 110 patients, 56.4% (n=62) were discharged, while 43.6% (n=48) died. The mortality rate was 38.6% among those who did not develop nephrotoxicity during AmB-d treatment, while 52.5% among those who developed AmB-d associated nephrotoxicity.

## Discussion

In recent years, AmB-d has fallen into disfavor in the treatment of fungal infections due to its untoward effects. In this regard, nephrotoxicity is the most important dose-limiting side effect associated with AmB-d use. Although the exact mechanisms of AmB-d associated nephrotoxicity remain obscure, studies suggest that renal vasoconstriction and tubular injury may play a major role [6,10,11]. The tubular injury also leads to hypokalemia and hypomagnesemia [6,10,11]. In a study by Walsh et al. comparing AmB-d and liposomal AmB, both drugs were associated with similar survival rates (93% vs. 90%) and less responses (58%, for each), while toxicities were less frequent in the liposomal AmB arm [8]. In another meta-analysis, the incidence of AmB-d-associated nephrotoxicity was higher (32.5% vs. 14.5%) [8,12]. However, lipid-based formulations and newer generation anti-fungal agents are associated with a higher economic burden due to their high acquisition costs. In one study, Roux et al. reported that most anti-fungal treatments for invasive candidiasis administered in patients admitted to intensive care units may actually be unnecessary, leading to increased treatment costs. Therefore, these authors recommended the use of AmB-d for the first-line treatment of such infections, based on similar efficacy with newer antifungal agents and lower treatment costs [13]. While lipid-based formulations have largely replaced AmB-d in countries with adequate resources, the conventional formulation continues to be widely utilized in developing countries, where cost-related issues remain a source of concern. However, when the new conditions of the world combined with the major burden of the pandemic on healthcare systems are taken into consideration, it is likely that cost will play a more important role in treatment decisions throughout the world, not only in the developing countries. Similarly, although the use of AmB-d is declining, it still remains a therapeutic option in certain indications in our country.

A generally reversible and temporary decrease in GFR has been reported in approximately 5% to 80% of patients receiving treatment with AmB-d [14,15]. The net effect involves an elevation of serum creatinine levels. In a nine-year-long retrospective study by Harbarth et al. involving 494 patients, nephrotoxicity of any degree was reported in 138 (28%) during AmB-d treatment, with 58 patients (12%) having moderately severe nephrotoxicity [14]. In another report from the Hematology-Oncology and Stem Cell Transplantation Unit of the Talegani Hospital, Tehran increased serum creatinine and BUN was reported in 9 of 35 patients (25.7%) during AmB-d treatment [15]. Khalili et al. observed acute kidney injury in 10 of 13 patients (76.92%) who were treated with AmB-d alone; however, the incidence of nephrotoxicity was 86.6% among those who also received ceftriaxone and/or vancomycin in combination with AmB-d [16]. In our study, the incidence of nephrotoxicity was evaluated in patients who only received the AmB-d formulation, and this figure was found to be 36.4% (n=40). Of these cases, 11.8% (n=13) and 3.7% (n=4) had stage II and III nephrotoxicity, respectively. The wide variation in the reported incidence of AmB-d-related nephrotoxicity may be due to differences in methodologies, clinical settings, related risk factors, and definitions used. The lower rates of nephrotoxicity in some studies may be related to the use of liposomal AmB, while higher rates in our study may be explained on the basis of the use of the conventional AmB only in our patient group.

Stage 3 kidney failure exclusively associated with the use of conventional AmB-d among patients with hematological malignancies is rare, and renal dysfunction is multifactorial in most cases. Other potential culprits include the extent of the fungal infection, the current status of the underlying hematologic malignancy, presence/absence of comorbid conditions, use of other potentially nephrotoxic agents, involvement of other infectious pathogens, sepsis, and septic shock. Potential risk factors identified in previous prospective or retrospective studies include male gender, an average daily AmB-d dose of 35 mg, cumulative dose of AmB-d exceeding 2 to 5 g, dehydration, concomitant use of diuretics or nephrotoxic agents (e.g., aminoglycosides, cyclosporine, foscarnet, cisplatin, and ifosfamide) or corticosteroids, and presence of kidney dysfunction at baseline [17-19]. It is generally recommended to avoid AmB-d treatment in the presence of two or more of the above-listed risk factors [14,20]. The reported incidence of nephrotoxicity in patients receiving two or more nephrotoxic agents is 41% [8]. AmB-d related nephrotoxicity is generally reversible upon discontinuation of treatment [21,22], although kidney dysfunction may recur if treatment is re-initiated [22]. Renal replacement therapy such as dialysis may be required in approximately 15% of the patients affected [20,23]. In the current study, AmB-d related nephrotoxicity improved without treatment in 47.5% of the affected patients, and no patients required dialysis. While some of our patients developed stage 1-2 renal failure, these subjects tolerated the treatment under close supervision and without discontinuation of AmB-d, and laboratory parameters did not worsen, and the event got better over time. Laboratory parameters were checked on a daily basis, and prompt treatment adjustments were done when required. Therefore, mild renal failure occurring after initiation of AmB-d may not always require treatment discontinuation. In younger patients with good performance status and no risk factors, a continuation of treatment may be considered, provided that the patient is closely monitored. Adverse events related to drugs represent a significant cause of morbidity and mortality in neutropenic patients with hematologic malignancies. In our study, toxicities other than nephrotoxicity were also related with regard to their association with AmB-d treatment. Among these, the most common ones included drug infusion reactions (21%), hypokalemia (70%), hypomagnesemia (58.2%), and hepatotoxicity (21%). Most of the infusion reactions (86%) occur in the first five minutes of infusion [6]. In a study of liposomal AmB-d, Roden et al. reported that almost more than 20% of their patients had infusion-related reactions [24]. Despite some studies reporting higher rates of infusion reactions with the use of AmB-d versus the liposomal formulation (52% vs. 21%) [8], we failed to observe such differences. Only patients using AmB-d were included in this study. No comparison was made with the liposomal formulation. The treatment was discontinued in patients who experienced severe infusion reactions despite premedication. Symptoms fully recovered with no persistent effects in all patients. Seventy percent of our patients had hypokalemia in association with the use of AmB-d, and this figure was similar to the reported incidence of 75% to 90% [25]. In a recent study from Iran examining liposomal AmB in hematology-oncology practice, the reported incidence was 45% [26]. Hypomagnesemia, which is generally mild, is a frequent side effect of AmB treatment secondary to renal loss of magnesium. Therefore, routine monitoring of serum magnesium levels is recommended during AmB treatment [27]. In some publications, the reported incidence of hypomagnesemia ranged between 15% and 100%, depending on the dose and formulation of AmB [28]. Severe hypokalemia and hypomagnesemia secondary to AmB use may lead to metabolic complications, rhabdomyolysis, and life-threatening arrhythmias [25]. Although hypokalemia and hypomagnesemia were not uncommon in our patient group, no life-threatening serious complications were observed. Patients were closely monitored with laboratory tests according to their clinical characteristics, and appropriate replacement therapy was given. AmB-d related hepatotoxicity is rarely reported in the literature [29]. Persat et al., in a group of 20 patients with leukemia, observed no hepatotoxicity with itraconazole alone, while in 11 of the 12 (92%) leukemic patients receiving itraconazole combined with AmB-d had significant elevations in their liver enzymes [30]. In our study, 21% of the patients also had elevated liver enzymes, although only one patient required discontinuation of treatment due to hepatotoxicity. Our analysis based on clinical characteristics of the patients did not suggest a causal link between the use of AmB-d and hepatotoxicity. It appears that the risk of hepatotoxicity is higher in the context of multi-drug therapy.

## Conclusions

In conclusion, nephrotoxicity was observed in approximately one-third of our patients (36.4%) during AmB-d therapy, which resolved spontaneously without specific intervention in almost half of these subjects (47.5%). The mortality rate in those with nephrotoxicity was higher than those without nephrotoxicity, although this finding should be interpreted with caution due to the retrospective design of our study precluding direct comparisons. Other potential factors associated with mortality include the extent of the fungal infection, underlying hematologic malignancy, presence of comorbid conditions, multi-drug therapy, involvement of other infectious pathogens, presence of sepsis or septic shock, and the need for treatment in frail and elderly patients. In this group of patients, it would be prudent to closely monitor indices of kidney functions (e.g., serum creatinine, urea, potassium, and magnesium) as well as liver enzymes, in addition to daily clinical assessments with vital signs and physical examination. Appropriate patient selection and expertise of the clinical team are the two most important determinants of a successful management plan. In our country and other developing countries, avoidance of AmB-d therapy on the basis of potential nephrotoxicity concerns in favor of costlier treatments may not represent an appropriate approach for a carefully selected group of patients. Therefore, we believe that AmB-d should remain a valid therapeutic option in these settings, provided that patients are carefully selected and effectively monitored.
